# Interferograms plotted with reference change value (RCV) may facilitate the management of hemolyzed samples

**DOI:** 10.5937/jomb0-31250

**Published:** 2022-02-02

**Authors:** Kamil Taha Uçar, Abdulkadir Çat, Alper Gümüş, Nilhan Nurlu

**Affiliations:** 1 Bilecik Public Health Laboratory, Bilecik, Turkey; 2 Istanbul Gaziosmanpasa Training and Research Hospital, Istanbul, Turkey; 3 Basaksehir State Hospital, Istanbul, Turkey

**Keywords:** hemolysis, interference, preanalytical phase, laboratory errors, reference change value, hemoliza, interferencija, preanalitička faza, laboratorijske greške, promene referntnih vrednosti

## Abstract

**Background:**

The European Federation of Clinical Chemistry and Laboratory Medicine (EFLM) Working Group for Preanalytical Phase (WG-PRE) have recommended an algorithm based on the reference change value (RCV) to evaluate hemolysis. We utilized this algorithm to analyze hemolysis-sensitive parameters.

**Methods:**

Two tubes of blood were collected from each of the 10 participants, one of which was subjected to mechanical trauma while the other was centrifuged directly. Subsequently, the samples were diluted with the participant's hemolyzed sample to obtain the desired hemoglobin concentrations (0, 1, 2, 4, 6, 8, and 10 g/L). ALT, AST, K, LDH, T. Bil tests were performed using Beckman Coulter AU680 analyzer. The analytical and clinical cut-offs were based on the biological variation for the allowable imprecision and RCV. The algorithms could report the values directly below the analytical cut-off or those between the analytical and clinical cut-offs with comments. If the change was above the clinical cut-off, the test was rejected. The linear regression was used for interferograms, and the hemoglobin concentrations corresponding to cut-offs were calculated via the interferograms.

**Results:**

The RCV was calculated as 29.6% for ALT. Therefore, ALT should be rejected in samples containing >5.9 g/L hemoglobin. The RCVs for AST, K, LDH, and T. Bil were calculated as 27.9%, 12.1%, 19.2%, and 61.2%, while the samples' hemoglobin concentrations for test rejection were 0.8, 1.6, 0.5, and 2.2 g/L, respectively.

**Conclusions:**

Algorithms prepared with RCV could provide evidence-based results and objectively manage hemolyzed samples.

## Abstract (sr)

**Uvod:**

Radna grupa za preanalitičku fazu (WG-PRE) Evropske Federacije za kliničku hemiju i laboratorijsku medicinu (EFLM) preporučila je algoritam koji se zasniva na promeni referentne vrednosti (RCV) za procenu hemolize. Mi smo koristili ovaj algoritam za analiziranje osetljivih hemolizovanih parametara.

**Metode:**

Uzete su dve epruvete krvi od svakog od 10 učesnika, jedna koja je bila predmet mehaničke traume a druga je direktno centrifugirana. Istovremeno uzorci su bili razblaženi sa hemolizovanim uzorcima učesnika da bi se dobile željene koncentracije hemoglobina (0, 1, 2, 4, 6, 8 i 190 g/L). Analiziranje ALT, AST, K, LDH, T. Bil je izve deno primenom Beckman Coulter AU680 analizatora. Analitičke i kliničke cut-off vrednosti bile su zasnovane na biološkoj varijaciji za dozvoljivu nepreciznost i RCV. Algo ritmi su odgovarali direktno vrednostima ispod kliničke cut-off ili one između analitičke i kliničke cut-off. Ako je pro mena bila iznad kliničke cut-off test je odbačen. Linearna regresija je korišćena na interferograme i koncentracije hemo globina koje su odgovarale cut-off vednostima izračunatih preko interferograma.

**Rezultati:**

RCV je izračunat kao 29,6% za ALT. Prema tome potrebno je odbaciti uzorke za ALT koji su imali >5,9 g/L hemoglobina. RCV za AST, K, LDH i T. Bil izračunati su kao 27,9%, 12,1%, 19,2% i 61,2%, dok su uzorci sa koncentracijom hemoglobina za odbacivanje testa bili 0,8, 1,6, 0,5 i 2,2 g/L.

**Zaključak:**

Algoritimi pripremljeni sa RCV mogu da obezbe rezultate zasnovane na dokazima i objektivno procene hemolizovane uzorke.

## Introduction

The International Organization for Standardization (ISO) has established that laboratory errors may arise at every point related to the laboratory in the process, from the test request to diagnosis and treatments interrelated to test results [1]. This comprehensive definition showed that the laboratory's responsibilities are not only restricted to analyze the test and report its results. Nowadays, when laboratory results are considered crucial in clinical decisions, laboratories have a significant role, directly or indirectly, in the diagnosis and treatment of patients in many aspects [2]. Thus, laboratory errors should be handled with a holistic approach, evaluated, and fixed [3].

Most of the errors evident in laboratories arise from the preanalytical process [4]. Notably, the most common problem in this process is that of inappropriate samples [5]. Among inappropriate samples, hemolyzed samples are the most common. Evaluation of all samples sent to the laboratory in a previous study, revealed that 2.2% of them were hemolyzed samples [6]. Among samples accepted from emergency departments, up to 30% have been reported to be hemolyzed [7]. Hemolysis can be in vivo and in vitro. In vivo hemolysis occurs during various diseases or treatments and constitutes only 2% of all hemolyzed samples accepted to the laboratory [8]. In vitro hemolysis is almost the sole cause of hemolyzed samples encountered in laboratories and is caused by the following: blood collection process, sample handling process, sample processing process, and individual differences [9].

Hemolysis can be evaluated using two methods. The first is the classical method based on the technician's visual assessment. The visual evaluation method is discouraged because of its subjectivity and low reproducibility. Even if the color indicator charts with varying degrees of hemolysis, sample photographs are used for evaluation; this method's reliability and sensitivity are relatively low [10] [11]. The second method uses hemolysis index (H-index), a tool that enables estimating the sample free hemoglobin (Hb) concentration using automated systems. The H-index can report the degree of hemolysis to both the laboratory specialist and physician. Therefore, it can enable the evaluation of the test results affected by hemolysis in the analyzed sample. Automated systems are reportedly more reliable than visual evaluation in determining hemolysis, and therefore, it is recommended for use instead of the visual method [12]. Currently, the management of hemolyzed samples involves applying four options according to the hemolysis level, as follows: analysis of the sample followed by reporting the result; reporting the result and its interpretation; rejection based on the test; thorough sample rejection [13]. For correct implementation, the serum index should be determined based on the test for the management of hemolysis.

The C56-A guideline of the Clinical and Laboratory Standards Institute (CLSI) recommends that interference studies should first be carried out by in vitro diagnostic (IVD) manufacturers and that the laboratories should design, as well as use, their algorithms. It also states that the reference change value (RCV) can be used as the allowable total error criterion for interference studies [14]. Fraser stated that when the interfering effect of the substance on the test result exceeds the RCV, it could change the actual level of the measured analytes, with a clinically significant difference [15]. The entire procedure was presented in an opinion letter that was recommended for clinical biochemistry tests published by the European Federation of Clinical Chemistry and Laboratory Medicine (EFLM) Working Group for Preanalytical Phase (WG-PRE) in 2018 [16]. The EFLM WG-PRE has proposed an algorithm in line with this data to evaluate hemolysis interference for clinical chemistry tests. Interferograms were created by adding the allowable analytical coefficient of variation (CV) values and RCV as evaluation criteria after graphs were drawn by calculating the percentage change according to the Hb concentration for each test. Recent studies recommend a new algorithm that allows the interpretation of the test result through interferograms [12].

In this study, we aimed to develop algorithms to be used in the alanine aminotransferase (ALT), aspartate aminotransferase (AST), lactate dehydrogenase (LDH), potassium (K), and total bilirubin (T. Bil) tests for our laboratory, with respect to these suggestions, and to compare the data obtained with the manufacturer's statements.

## Materials and Methods

This study was performed at the Medical Biochemistry Laboratory of the Gaziosmanpasa Training and Research Hospital, with the approval of the local ethics committee (decision date & number: April 10, 2019-56), in accordance with the Declaration of Helsinki. The study was explained to all volunteers and then informed consent was obtained.

### Study design and sample preparation procedure

The sample size was determined to be 10 people, as per the CLSI EP 07A2 guideline. Acute or chronic disease, regular medication use, bleeding disorder, and pregnancy were selected as the exclusion criteria. Two tubes of blood were collected from 10 apparently healthy volunteers aged between 18 and 50 years. A single experienced phlebotomist was appointed to perform blood collection, to avoid hemolysis caused by phlebotomy. The samples were collected in the collection tubes (BD Barricor 5.0 mL, 13x100 mm, Becton, Dickenson and Company, NJ, USA) using the blood collection device (BD Vacutainer Holder, Becton, Dickenson and Company, NJ, USA) equipped with a needle (BD Vacutainer Eclipse 21G, BD-Belliver Industrial Estate, Plymouth PL6 7BP, UK).

One of the paired tubes was centrifuged without anyintervention to obtain the hemolysis-free samples. Theblood from the other tube was passed through the needle10 times using an injector to achieve mechanical hemolysis [12]. The mechanical trauma method was preferred toobtain samples similar to hemolyzed samples sent to thelaboratory. This method ensured that the hemolyzed sample contains leukocytes and thrombocytes [13]. Subsequently, the tubes were centrifuged at 2000 × g for 10min following the manufacturer’s recommendation.

**Figure 1:**
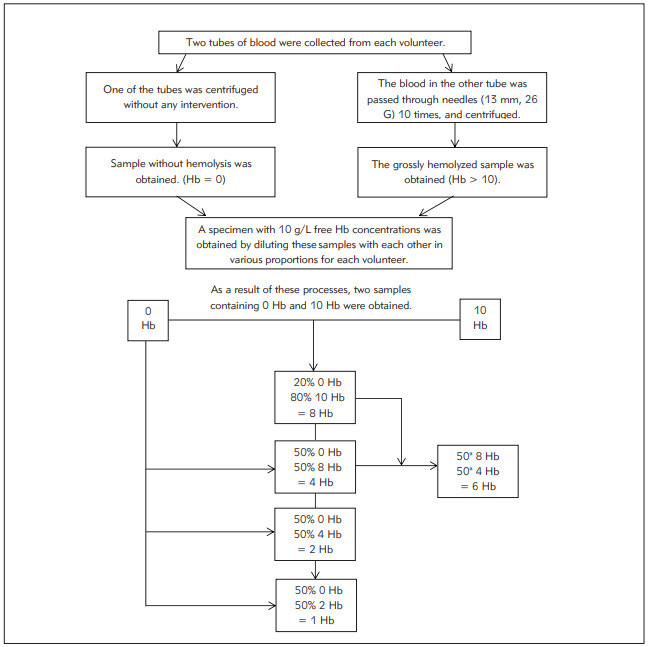
Preparation of hemolyzed samples The samples were prepared individually for each volunteer (n=10). As aresult, samples with the desired Hb concentrations (0, 1, 2, 4, 6, 8, and 10 g/L) were procured. Hb was expressed as g/L

Free Hb concentrations were measured in the separated plasma samples by the spectrophotometric methodusing the auto hematology analyzer (Mindray BC 6800, Shenzhen Mindray Bio-Medical Electronics Co., China;imprecision of Hb: 1.0%). It was confirmed that one of thesamples had an Hb concentration of 0 g/L, while the otherhad a Hb concentration >10 g/L. To obtain samples withthe desired free Hb concentrations (1, 2, 4, 6, 8, and 10g/L), the hemolyzed samples were diluted with the hemolysis-free sample of the same volunteer. The samples wereprepared individually for each volunteer, thus preventingdilution bias and inter-individual differences from affectingthe values obtained. After obtaining the diluted samples,each sample was analyzed duplicate on the Mindray BC6800 analyzer to confirm the desired Hb concentration.The process for sample preparation is shown in Figure 1.

### Evaluated tests and reagents

ALT, AST, LDH, K, and T. Bil tests were performed ona Beckman Coulter clinical chemistry analyzer AU680(Beckman Coulter, Brea, CA). Tests were analyzed in duplicate for each sample. The manufacturer’s original reagentswere used in this study. The principles of the tests havebeen elaborated in the subsequent text.

• ALT: IFCC (without pyridoxal phosphate activation)

• AST: IFCC (without pyridoxal phosphate activation)

• LDH: Lactate to pyruvate, IFCC

• K: Ion-selective electrodes, indirect

• T. Bil: 3,5-dichlorophenyl-diazonium tetrafluoro -borate (DPD) with caffeine, and a surfactant

In the technical sheets of reagents, it has been reported that hemolyzed samples should not be used for AST, K,and LDH tests. No Hb concentration, H-index limit, or biasof interference has been specified. It has been reported thatthe interference could be up to 10% in samples with 5 g/Lfree Hb for ALT and 0.45 g/L free Hb for T. Bil. H-indexvalues are expressed qualitatively on the Beckmann AU680analyzer. The cut-off degrees and the corresponding approximate Hb concentration ranges are presented in Table 1.

**Table 1:** The cut-off degrees and the corresponding approximate hemoglobin (Hb) concentration ranges in BeckmannCoulter AU680 analyzer

Cut-offs specified by the manufacturer	Approximate free Hb concentration ranges (g/L)
0	Hb ≤ 0.5
+	0.5 <Hb ≤ 1
++	1 < Hb ≤ 2
+++	2 < Hb ≤ 3
++++	3 < Hb ≤ 5
+++++	5 < Hb

### Statistical analysis

The percentage difference was calculated for the samples from each patient. Next, the mean percentage differences were calculated for each Hb concentration using thepercentage differences for 10 different volunteers. The Shapiro–Wilk test was used to evaluate whether the valueswere normally distributed. All values in our study were distributed normally, and the results are presented as the mean ± SD. Subsequently, interferograms were plotted for theevaluated tests. The scatter plots and linear regressionmethod were used when creating interferograms, and theHb concentrations corresponding to analytical and clinical cut-offs were calculated using the regression equation of thegraphs. Analytical cut-offs were determined according to thedesirable allowable CV (analytically acceptable imprecision (I%)) values of the biological variation (BV) databases. Clinicalcut-offs were determined according to the RCV. Intra-individual CV (CVI) for tests was obtained from the EFLM BV database and Westgard Desirable BV database [17] [18]. The Hbconcentration for sample rejection was determined to be10 g/L following the recommendation of EFLM [16]. The formulas for the calculation are as follows:


(1) \text{Percent difference} (\%) = ( \frac{\text{Result of hemolyzed sample - Result of nonhemolyzed sample}}{\text{Result of nonhemolyzed sample}} ) \times 100 \\
\\ I(\%) = 0,5 \times CV_{I}\\ RCV(\%) = \sqrt{2} \times 1,96 \times \sqrt{(CV_A^2 + CV_I^2)}

I: Analytically acceptable imprecision

CV_A_: Laboratory analytical CV

CVI: Within-subject BV

For laboratory analytical CV (CV_A_) calculation, twolevels of internal quality controls (Beckman Coulter ControlSerum 1–2, Inc., USA), which were run on 20 differentdays, were used. The following formula was used for calculation [16]:


(2) }{}CV_{A} = \frac{\text{QC Level 1} \text{CV}_{A} + \text{QC Level 2} \text{CV}_{A} }{2}

The calculations were performed, and interferogramswere plotted, using MedCalc® Statistical Software version19.6.4 (MedCalc Software Ltd, Ostend, Belgium) andMicro soft Office 365 (Microsoft Excel Software, MicrosoftCorporation, US).

## Results

**Figure 2:**
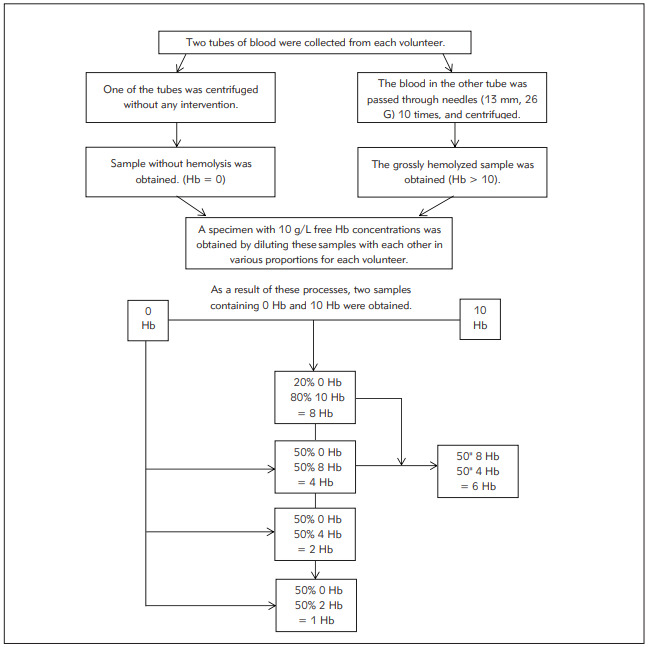
Interferograms of the evaluated tests Analytical cut-off: desirable allowable imprecision from the biological variationdatabases, clinical cut-off: reference change value (RCV). Regression equations and coefficient of determinations (R2) are presented in the corners of the figures. The regression line represents the 95% CI. Hb: Hemoglobin, ALT: Alanine aminotransferase,AST: Aspartate aminotransferase, K: Potassium, LDH: Lactate dehydrogenase, T. Bil: Total bilirubin.

According to the specified tests using samples with determined Hb concentrations, mean percentage differences (%) between the samples are presented in Table 2. The interferograms of the tests are shown in Figure 2. The desirable allowable impression (I%)-analytical cut-off for ALT was obtained as 5.0%. The RCV - clinical cut-off was calculated as 29.6% (Table 3). Hb concentrations corresponding to these values were calculated as 0.8 and 5.9 g/L based on the regression equation (Table 3). Accordingly, for samples with Hb concentration <0.8 g/L, ALT results could be reported directly; with 0.8-5.9 g/L, ALT results could be reported with interpretation (Table 3). Since the percentage change values of ALT in samples with Hb >5.9 g/L were greater than the RCV, it was deemed appropriate for rejection (Table 3). For AST, LDH, K, and T.Bil, I% values and RCVs were found to be 4.8%, 27.9%; 2%, 12.1%; 2.6%, 19.2%; and 10.9%, 61.2%, respectively (Table 3). The Hb concentration cut-offs obtained from the regression equation for the four tests were found to be 0.2, 0.8 g/L; 0.2, 1.6 g/L; 0.3, 0.5 g/L, and 0.5, 2.2 g/L; respectively (Table 3). The H-index measurement results in each hemolyzed sample were observed in agreement with the manufacturer's H-index cut-offs. However, it was observed that the ratio of change in analyte results due to hemolysis did not fully comply with the manufacturer's statements.

**Table 2:** Mean percentage differences (%) between samples from each subject (n=10) for determined hemoglobin (Hb) values,according to the specified tests All values are presented as mean ± SD (%). Normal distribution of values was proved by Shapiro-Wilk test (p > 0.05)

Tests	Hb = 1 g/L	Hb = 2 g/L	Hb =4 g/L	Hb = 6 g/L	Hb = 8 g/L	Hb = 10 g/L
Alanine aminotransferase, U/L	6.7 ± 3.5	11.4 ± 4.2	19.6 ± 7.6	31.0 ± 12.3	39.6 ± 15.6	48.2 ± 15.3
Aspartate aminotransferase, U/L	46.1 ± 11.3	83.2 ± 17.9	162.9 ± 37.2	253.8 ± 58.3	354.8 ± 86.4	458.3 ± 92.6
Lactate dehydrogenase, U/L	50.7 ± 24.9	126.9 ± 45.3	287.7 ± 83.3	517.1 ± 102.0	659.6 ± 152.7	794.3 ± 163.1
Potassium, mmol/L	8.0 ± 4.7	15.3 ± 5.1	29.3 ± 7.8	47.3 ± 7.5	61.0 ± 10.8	74.3 ± 12.5
Total Bilirubin, mmol/L	-20.3 ± 12.6	-51.9 ± 22.5	-115.4 ± 45.5	-181.3 ± 78.6	-247.1 ± 101.2	-295.2 ± 110.5

**Table 3:** Regression equations (95% CI), R^2^, CV_A_ and CV_I_ values (%) of the evaluated tests. Analytical and clinical cut-off values(%) with corresponding free hemoglobin (Hb) concentrations calculated via regression equations. ^a^ Corresponding to analytical cut-off ^b^Corresponding to clinical cut-off. CI: Confidence interval, R^2^: Coefficient of determination, CV_A_: Analytical CV of the laboratory, CV_I_: Within – subject Biological Variation, Analytical cut-off: Desirable allowable imprecision values from Biological Variation databases, Clinical cut-off: RCV (Reference change value). ALT: Alanine aminotransferase, AST: Aspartate aminotransferase, K: Potassium, LDH: Lactate dehydrogenase, T.Bil: Total bilirubin

Tests	Regression equation<br>(95% CI)	R^2^	CV_A_<br>(%)	CV_I_<br>(%)	Desirable imprecision<br>(Analytical cut-off - %)	Hb^a^<br>(g/L)	RCV (Clinical cut-off - %)	Hb^b^<br>(g/L)
ALT	y = 1.22 (-0.33 – 2.76) + 4.77 (4.50 – 5.05) x	0.99	3.5	10.1	5.0	0.8	29.6	5.9
AST	y = -6.31 (-22.25 – 9.63) + 45.27 (42.43 – 48.11) x	0.99	3.0	9.6	4.8	0.2	27.9	0.8
K	y = 0.30 (-1.51 – 2.11) + 7.51 (7.19 – 7.84) x	0.99	1.5	4.1	2.0	0.2	12.1	1.6
LDH	y = -21.63 (-63.39 – 20.13) + 83.48 (76.05 – 90.91) x	0.99	4.6	5.2	2.6	0.3	19.2	0.5
T. BIL	y = -5.63 (-14.83 – 3.57) + 30.66 (29.03 – 32.30) x	0.99	3.4	21.8	10.9	0.5	61.2	2.2

**Figure 3:**
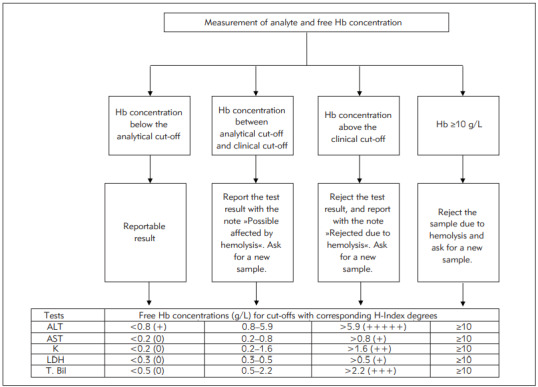
Basic presentation of the composed algorithms Hb concentrations corresponding to cut-offs were calculated via theregression equation. H-index degrees were adapted to the results in line with the cut-offs of the manufacturer. Hb: Hemoglobin,ALT: Alanine aminotransferase, AST: Aspartate aminotransferase, K: Potassium, LDH: Lactate dehydrogenase, T. Bil: Total bilirubin.

The CV_A_, CV_I_, analytical cut-off (I) and clinical cut-off values (RCV), regression equations (95% CI), coefficient of determinations (R^2^), and the corresponding Hb levels to cut-off values are presented in Table 3. A simple presentation of the algorithms is shown in Figure 3.

## Discussion

In our study, the hemolysis interference was evaluated for five clinical biochemistry parameters, based on the BV desirable I% (analytical cut-off) and RCV% (clinical cut-off). Accordingly, to determine the extent to which the tests were affected by hemolysis, LDH, AST, K, T. Bil, and ALT tests were performed, with respect to Hb concentration ranging from low to high.

For LDH, if the Hb concentration of the sample was <0.3 g/L, the test result could be reported directly; moreover, it could be reported with comment for 0.3 - 0.5 g/L Hb, and the test had to be rejected for >0.5 g/L Hb. For AST and K, the corresponding Hb values (in g/L) were found to be <0.2, 0.2 - 0.8, and >0.8, and <0.2, 0.2 - 1.6, and >1.6, respectively (Figure 3). As per the manufacturer's recommendation, these three tests should be rejected on the hemolyzed samples. As the initial cut-off value determined by the manufacturer for the H-index is 0.5 g/L, it can be assumed that the hemolyzed sample was meant for samples containing 0.5 g/L Hb. In another study, the upper limit of Hb reference value for hemolysis-free plasma samples was found to be 0.13 and 0.10 g/L in two different biochemistry analyzers [19]. Although no information is available in the literature for our device, these values may be considered valid for our study, since it involved working with plasma samples containing lithium heparin. In fact, the Hb concentrations corresponding to the analytical cut-offs we calculated in our study for LDH, AST, and K seem close to these values. Besides, the tests most affected by hemolysis were those of LDH, AST, and K. However, our findings - particularly those associated with K - do not seem entirely consistent with the manufacturer's statement. It has been observed that K can be analyzed on samples containing up to 1.6 g/L Hb.

When the Hb concentration of the sample for T. Bil was <0.5 g/L, the result could be reported directly. It was observed that for 0.5 - 2.2. g/L Hb, the result could be reported with comment and that it should be rejected for >2.2 g/L Hb. For ALT, these Hb values were found to be <0.8, 0.9 - 5.9, and >5.9, respectively (Figure 3). The manufacturer stated that the hemolysis interference could be less than 10% in samples containing Hb up to 0.45 g/L for T. Bil and 5 g/L for ALT. Although this information is consistent with our findings, it is insufficient to manage samples containing 0.45 and 5 g/L in the T. Bil and ALT, respectively. According to our findings, samples containing up to 2.2 g/L and 5.9 g/L could be analyzed for T. Bil and ALT, respectively, and the result could be reported with the interpretation.

Perovic and Dolvic [20] evaluated the hemolysis levels of 25 clinical biochemistry parameters using RCV and compared the results with the manufacturer's statements. Similar to our study, the Beckman Coulter AU480 clinical chemistry analyzer and reagents from the same manufacturer were used in this study. It was observed that the findings within the reference range obtained in this study were consistent with our findings, and in the same study, it was stated that the manufacturer's statements were insufficient for ALT tests, among the tests evaluated. Studies on other devices and reagents have also obtained results that do not comply with the manufacturer's declarations [21] [22] [23] [24]. On the other hand, the general approach assumes the significant change affecting the result as ± 10% and presents the cut-off values accordingly. However, this ratio is far from being a proper criterion for every test [24]. Using RCV or other criteria instead of±10% change value as a standard for each test can also support their flexibility [24] [25]. Additionally, rejection rates may decrease by accepting RCV as a cut-off for test rejection [23]. Based on these data, the use of RCV and these algorithms by manufacturers while performing interference studies will make the H-Index more beneficial.

Moreover, several problems have been observed with the routine use of the H-index. EFLM WG-PRE had a call for IVD manufacturers to provide more clarity, with respect to serum indexes, in 2018 [26]. One problem is that the Hindex is reported as degrees in some devices and free Hb concentrations in other devices. EFLM WG-PRE recommended a harmonization in reporting results via the use of a common unit, free Hb (g/L). Another problem is that manufacturers do not adequately report the interference specifications of the kits according to their H-index. For laboratories, inadequate information could be a significant problem in the management of hemolyzed samples. This situation was also evaluated in a large survey study conducted by EFLM WG-PRE, with the participation of 1405 laboratories in 37 European countries. It has been reported that many laboratories indicate heterogeneity of data on interference as the reason for avoiding serum indexes, and 67% of laboratories using serum indexes use the cut-off values recommended by the manufacturer without verification [27] [28]. On the other hand, EFLM WG-PRE assumes that IVD producers did not fully comply with CLSI guidelines while performing interference studies; therefore, verification studies should be carried out by the laboratories [28]. Furthermore, internal, and external quality control evaluations for H-index are recommended, given the critical importance of H-index results in the evaluation of other test results [26]. It is thought that the approaches in which preanalytical variables are considered, especially in externalquality control assessments, can provide efficient use of the H-index [12].

Our findings support this point of view. As seen in our study, it is thought that presenting the hemolysis interference with the qualitative cut-offs given based on approximate concentrations recommended by the manufacturer instead of the free Hb concentrations may be unsatisfactory for the evaluation and interpretation of hemolysis. Moreover, it would be more beneficial to use interferograms, instead of a single cut-off value, for managing hemolyzed samples. Interferograms provide more precise information for testing or sample rejection; hence, they allow laboratories the flexibility to accept the sample, and analyze or reject the tests. It is necessary to be careful when deciding to reject the samples that have been submitted to the laboratory. The decision on which sample is inappropriate, decision on tests that cannot be carried out in these samples, or decision to reject the sample should be based on evidence-based information. The blood sample is submitted to laboratories with the intent to elucidate crucial information about the patient being examined. The importance of correct interpretation of the test results, and their subsequent reporting, by a laboratory professional is analogous to that of accurate interpretation of a physical examination by a physician [29]. The laboratory specialist's process for extensive assessment of the sample also includes the determination of which sample is suitable for analyzing or the determination of which test will be carried out. Analyzing the samples and reporting the correct result should be one of the priorities of laboratories due to the prolonged diagnostic process, additional cost, and other problems related to patient/doctor safety that may arise due to incorrect results and rejection [16]. When we collectively evaluate these findings and opinions, the use of H-index and other serum indexes with interferograms appears to be beneficial for laboratories.

There are some limitations to this study. First, only five parameters affected by hemolysis were selected. A study including all clinical biochemistry tests would yield a more comprehensive result. Second, the reference method for Hb measurement is the hemoglobin cyanide method measured spectrophotometrically [30]. However, the Mindray BC 6800 auto hematology analyzer used in our routine laboratory was used in our study to measure the Hb value. It is reported in the literature that the device we use is satisfactory in terms of analytical performance for Hb measurement [31]. Nevertheless, using the reference method could provide more accurate results. Finally, the test results we evaluated in our study are within the reference ranges valid for the relevant test. Conducting evaluations in concentrations exceeding the reference range may help use the Hindex more effectively.

Therefore, using the H-index with RCV and adapting the information to interferograms could be advantageous for laboratories to identify and manage hemolyzed samples. Single cut-off values are not suitable for use in interferograms alone. As observed in our study, it seems more appropriate to perform local studies to verify these values and determine the analytical and clinical cut-offs.

## Dodatak

### *Research funding*:

This research did not receive any specific grant from funding agencies in the public, commercial, or not-for-profit sectors.

### Conflict of interest statement

All the authors declare that they have no conflict of interest in this work.
